# Successes and Failures of Static Aptamer-Target 3D Docking Models

**DOI:** 10.3390/ijms232214410

**Published:** 2022-11-19

**Authors:** John G. Bruno

**Affiliations:** Nanohmics Inc., Austin, TX 78741, USA; jbruno@nanohmics.com

**Keywords:** aptamer, Avogadro, beacon, FRET, PatchDock, RNA Composer, YASARA

## Abstract

While Molecular Dynamics simulation programs are probably superior for predicting the binding and affinity of aptamers and their cognate ligands, such molecular dynamics programs require more computing power and analysis time than static docking programs that are more widely accessible to the scientific community on the internet. Static docking programs can be used to investigate the geometric fit of rigid DNA or RNA aptamer 3D structures and their ligands to aid in predicting the relative affinities and cross-reactivity of various potential ligands. Herein, the author describes when such static 3D docking analysis has worked well to produce useful predictions or confirmation of high-affinity aptamer interactions or successful aptamer beacon behavior and when it has not worked well. The analysis of why failures may occur with static 3D computer models is also discussed.

## 1. Introduction

In the past, protein (e.g., antibody or other) receptor–ligand docking model generation has been relegated to only a small number of laboratories with high-powered computing capabilities especially for Molecular Dynamics (MD) and very few groups dealt with nucleic acid aptamer structures for docking analyses. However, with the increasing popularity of aptamers, at least as research reagents, the desire to better model and understand their 3D structures and binding characteristics is increasing dramatically and there is more demand for at least superficial knowledge of how aptamers and their ligands may interact and fit together to aid in the optimization of binding [1,2,3,4,5,6,7,8,9,10].

**Figure 1:**
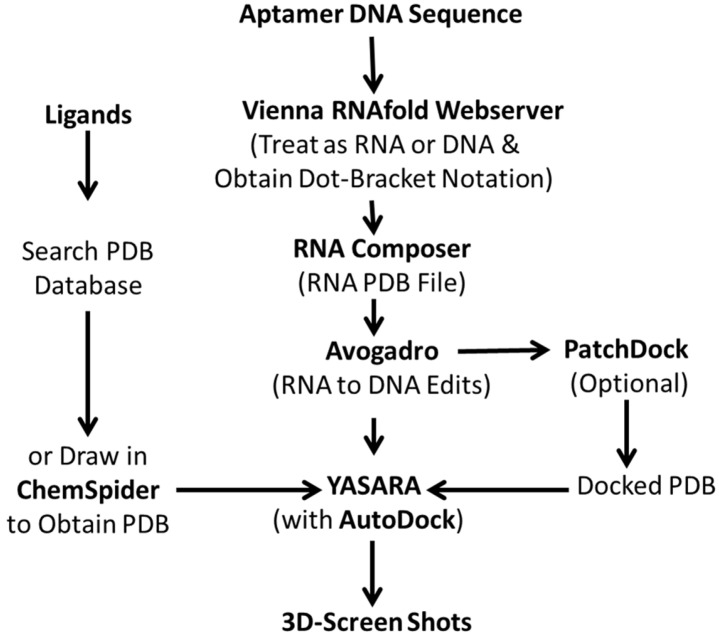
A generalized algorithm used by Bruno and others [16,17,18,19,20] for piecing together free internet software to generate static 3D aptamer structures and docking models of aptamers bound to their cognate ligands.

While it is true that MD packages such as GROMACS (Groningen MAchine for Chemical Simulation) [11,12,13] are now free, available on the internet and becoming more accessible via Amazon Web Services’ (AWS) supercomputer access, many researchers with aptamer modeling needs still tend to be bewildered and daunted by the technology and shy away from its use. Indeed, the author himself was reluctant to delve into this area a decade ago because it seemed so complex and laden with unforeseeable inaccuracies. However, the need for high-quality aptamer 3D structures to modify aptamer binding affinities and specificities plus the high cost of subcontracting the aptamer 3D docking analyses [14,15] drove the author to do it himself and devise an algorithm that combined a number of extant internet sites and programs to produce static 3D models of aptamers and their ligands (i.e., Figure 1). Admittedly, this approach is somewhat labor-intensive and probably inferior to MD analyses on supercomputers that account for vibrations, force fields, geometries and free-energy minimization upon binding, hydration, ionic strength, etc. However, the author’s simpler “do-it-yourself” (DIY) approach [16,17,18,19] at least allows a basic understanding of aptamer–ligand interactions upon which one could perhaps begin to optimize binding [15,16]. In addition, the author’s static DIY approach seemed less psychologically daunting to some researchers who developed it or similar approaches independently [20]. 

The author’s DIY or homemade aptamer structure determination and docking model algorithm were similarly developed in parallel by the iGEM INSA (Institut National Des Sciences Appliquées) group in Lyon, France (https://2016.igem.org/Team:INSA-Lyon/Software, accessed on 15 November 2022) who reasoned that they could use the Vienna RNA secondary structure package followed by use of Rosetta to develop a 3D model of the folded RNA version of an aptamer (the author and Rock et al. [17,18] used RNA Composer from Poland) which is then converted to DNA using a chemical editing program such as Avogadro. However, as the reader shall see, this may be a flaw in logic for both INSA and the author, because one is not truly computing the folding of the DNA aptamer, but its RNA surrogate followed by conversion back to DNA post-folding. The remainder of this paper tests the hypothesis that one can fold the aptamer as an RNA structure and then analyze its binding characteristics correctly by simply converting it back to DNA to make binding optimization and fluorescence quenching or dequenching predictions. Of course, if one can stop at the 3D RNA structure and use an RNA aptamer, this approach is probably entirely valid. However, the rather artificial conversion back to DNA instead of folding the DNA version of the aptamer itself is a dubious step that could lead to inaccurate results and predictions as discussed herein. 

## 2. Static 3D Docking and Analysis Algorithms

The basic algorithmic workflow used by Bruno [16,17,18,19] and others [20] to develop static DNA or RNA aptamer 3D docking models with their respective cognate ligands is shown in Figure 1 below. The algorithm is rather arduous, but free of cost and widely available to the public with little training for those willing to patch together inputs and outputs from various internet software programs. 

The process starts with the aptamer DNA sequence of interest, although an RNA aptamer is clearly easier to start with to model. However, for DNA, the sequence is processed through Vienna RNA’s RNAfold webserver (http://rna.tbi.univie.ac.at/cgi-bin/RNAWebSuite/RNAfold.cgi, accessed on 15 November 2022) using the advanced option for DNA parameters (Matthews model, 2004 should be selected). The user can choose the temperature for modeling and the author chooses 25 °C for diagnostic applications and 37 °C for therapeutic in vivo aptamer modeling because the author has seen significant temperature-dependent differences in 2D and 3D structures. As part of its output, the Vienna RNA RNAfold webserver then outputs dot-bracket notation that can be input to RNA Composer software at Poznan University of Technology in Poland (https://rnacomposer.cs.put.poznan.pl/, accessed on 15 November 2022 [21]) which in turn generates a 3D RNA model of the original DNA aptamer sequence. 

**Figure 2:**
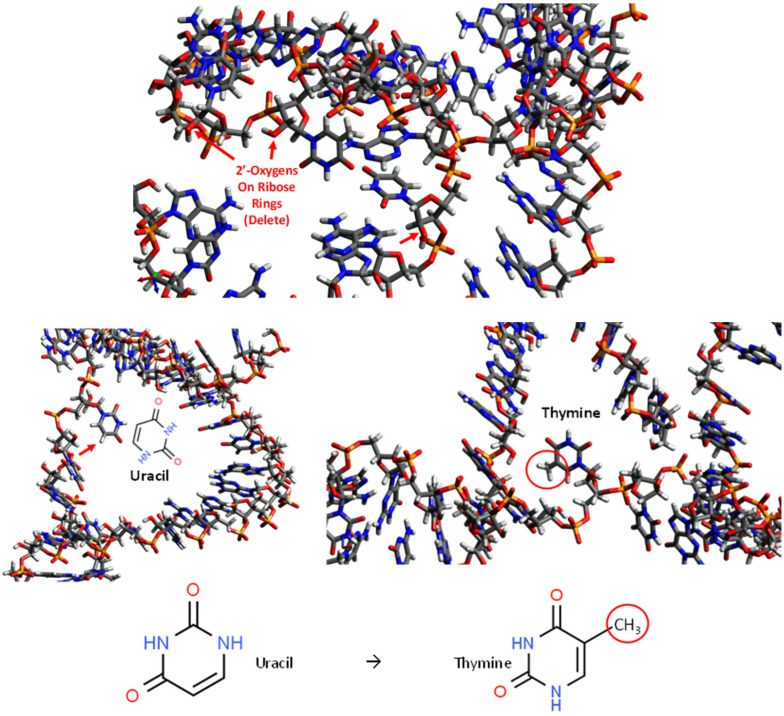
Graphics from Avogadro to illustrate the tedious and error-prone nature of converting generic 3D RNA aptamer structures into their DNA counterparts. The process includes deleting all 2′ hydroxyl groups on ribose rings at the 2′ oxygen atoms (top panel) and addition of methyl groups to all uracil rings (red arrow at lower left panel) as shown to convert them to thymines (red circled methyl group on thymine lower right panel).

The next step is the most tedious and may be a source of ultimate fatal errors in this sort of static 3D aptamer modeling. It is a manual modification of the RNA version of the original DNA aptamer sequence back into a DNA molecule using Avogadro software. Figure 2 illustrates how Avogadro is used to painstakingly review each nucleotide to ensure that: (1) all 2′ hydroxyl (red and white “elbow” structures in Figure 2) on the ribose sugars are identified and deleted and (2) all uracil molecules are converted to thymines by addition of methyl groups as shown in the figure. Figure 2 somewhat conveys the complexity of this process which can be error-prone, especially for the novice leading to the wrong 3D aptamer model, if one misses a 2′ hydroxyl deletion or uracil to thymine conversion. However, even more importantly, converting back to DNA from the folded RNA structure is an “artificial” tactic (i.e., the aptamer was not folded as a DNA molecule, but as its RNA surrogate and then converted back to its presumed DNA structure). What is needed to rectify these concerns is a software program that minimizes the free energy of DNA to accurately and directly predict its 3D structures. Of course, what is ultimately needed to validate any static models is X-ray crystallography and diffraction studies of the bound aptamer–ligand crystals.

## 3. Published Examples

### 3.1. Successes

In this section, several published successes produced from static 3D aptamer modeling will be cited, but the inclusion of all the associated graphics is not truly feasible. Therefore, the reader is referred to specific literature in each section to view the specific aptamer–ligand models. 

#### 3.1.1. Methylphosphonic Acid (MPA) Aptamer Beacon

MPA is the core of a number of organophosphorus (OP) nerve agents such as sarin, soman and VX. The author was funded by the U.S. Defense Department to develop a competitive aptamer beacon to detect MPA and successfully did so, by first coupling amino-MPA to tosyl-magnetic microbeads and using those particles to probe a SELEX random DNA library for the highest affinity DNA oligonucleotide binders. The end result was a high-affinity conserved sequence, namely TTTAGT, which the Southwest Research Foundation (San Antonio, TX, USA) confirmed using GROMACs MD software [15]. The author was later funded to develop aptamers specifically against a derivative of soman using the same sort of analyte-coated magnetic bead SELEX approach and the same TTTAGT segment again emerged in the final consensus aptamer pool [16]. The associated static 3D modeling strongly suggested that the TTTAGT segment energetically favored sliding past the peripheral or flanking chemical moieties onto MPA itself with high affinity which was supported by Enzyme-Linked Aptamer Sorbent (ELASA) microplate absorbance data [22].

#### 3.1.2. C-Telopeptide (CTx) Bone Marker Aptamer Beacon

The author was also funded by the U.S. National Aeronautics and Space Administration (NASA) to develop aptamer beacons for monitoring biomarkers of astronaut bone loss on lengthy space missions [14,23]. The author developed aptamers against CTx from bone collagen and used both GROMACs and static 3D aptamer models to reliably determine the optimal binding loops for beacon development [11], which led to low nanogram per ml detection of CTx even in desalted urine using a handheld fluorometer. 

#### 3.1.3. Hydroxyoctadecadieneoic Acids (HODEs) Docked with Analgesic Aptamers

**Figure 3:**
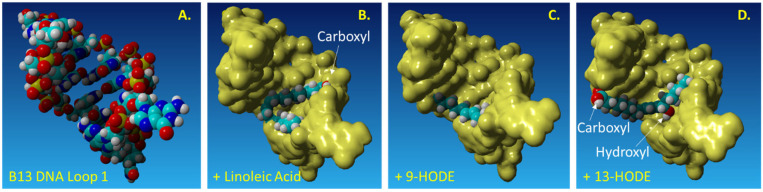
(**A**–**D**) A truncated aptamer loop designated B13 was developed against 13-HODE, but clearly also swallows the 9-HODE isomer and linoleic acid precursor as well when docked using PatchDock and visualized with YASARA to account for its dominant heat and burn pain analgesia in a rat model (i.e., the truncated B13 aptamer loop is capable of binding and blocking linoleic acid and both of its oxidized pain-inducing products). YASARA stands for “Yet Another Scientific Artificial Reality Application” 3D molecular modeling software.

The author was funded by the U.S. National Institutes of Health (NIH) to develop aptamers to bind HODEs which are known to induce heat and burn pain from oxidized linoleic acid. The aptamers were designed to be non-addictive injectable analgesics and they, indeed, proved to significantly delay rat hind limb retraction from an uncomfortably heated block (U.S. Patent No. 10,100,317 and [16,24]). In the initial 3D static ball and stick modeling, one could see tighter binding (no spatial gaps) between the 13-HODE isomer versus the 9-HODE variant by the cognate B13 aptamer loop shown in Figure 3A which correlated with a slight preference for the 13-HODE over the 9-HODE in ELASA cross-reactivity assays [16,24]. However, subsequent 3D surface modeling shown in Figure 3 suggests that both the 9- and 13-HODE isomers as well as their linoleic acid precursor are essentially engulfed by the aptamer loop which probably accounts for its significant analgesia in the rat pain assessment model because it is able to bind both the linoleic acid precursor substrate and both oxidized pain-inducing HODE products rather tightly. All three ligand–aptamer combinations shown in Figure 3B–D gave very similar shape complementarity scores ranging from 3818–4170 (a small variation in scores) in PatchDock indicating that they are of about equal affinity as corroborated by ELASA.

#### 3.1.4. Brain Natriuretic Peptide (BNP) Aptamer Sandwich Assay

The author began to truly formalize the 3D modeling algorithm from available free internet website software integration as shown in Figure 1 using an aptamer sandwich assay that the author knew worked quite well. In particular, the BNP aptamer sandwich assay on the surface of magnetic microbeads produced low picogram per ml detection limits via electrochemiluminescence or ECL [25]. This high affinity was corroborated by 3D static docking models that showed no space at all between BNP and its capture and reporter aptamers [25]. Indeed the capture and reporter aptamers formed an actual tightly fitted “cup” for the BNP ligand to sit in. 

#### 3.1.5. Prostate-Specific Antigen (PSA) Variant Modified Aptamer Binding

In silico static modeling of exotic base substitutions may be the most pragmatic and important use of static 3D docking models to serve as at least a preliminary “first pass” for studying the potential impact of exotic bases in aptamer structures on their binding affinity and specificity. Perhaps the greatest success of static 3D modeling performed by the author to date has been the addition of unnatural diaminopurine (DAP) into the putative binding sites of the author’s best PSA aptamer to add an additional hydrogen bond that appears to increase the specificity of the PSA aptamer several fold for a particular variant that was previously undetectable by immunoassays [16]. In particular, in PSA isoleucine (I) and threonine (T) genetic variants exist at position 179 [19]. Bruno’s highest affinity unmodified aptamer candidate could not discriminate I and T variants [19]. Thus, Bruno used 3-D YASARA docking models to reveal that a particular adenine in the candidate aptamer is proximal to the I and T locations PSA. Bruno then reasoned that the substitution of a DAP for the adenine proximal to I or T would either lead to an additional hydrogen bond (strong attractive force) or result in an additional repulsive force between the extra amine group’s electron pair in DAP and the highly electronegative polarized oxygen in the hydroxyl group of threonine, thus providing a means for discrimination and greater selectivity [19]. This approach only produced about a 20% greater binding of the modified aptamer to the PSA I-variant by ELASA as previously published [19], but it demonstrated the aptamer-PSA binding was perturbed in that region leading to a difference in the binding capacity that could be modeled with 3D docking software (YASARA).

### 3.2. Failures 

Despite some successes already cited in Section 3.1.1 and Section 3.1.2 for CTx and MPA aptamer beacons (a form of Fluorescence Resonance Energy Transfer or FRET) and intensive study of 3D docked aptamer–ligand models, the most common failure of rigid molecular modeling appears to involve FRET and the related fluorophore-quencher aptamer beacons. If static 3D modeling fails, it most often appears to fail when FRET is involved, perhaps because the margin of error for the very short (<100 Å) Förster distance is so small. The following subsections describe some seemingly inexplicable failures of static 3D docked models in which the aptamers and targets appear quite tightly bound even within the small Förster distance, so the design of a beacon or FRET system should be child’s play, but still failed to produce either a “lights on” or “lights off” fluorescence response as a function of analyte concentration.

#### 3.2.1. Cytomegalovirus (CMV) Antibody Aptamer Beacon

In another case, the author developed aptamers to act as simulants of CMV surface proteins for binding to human CMV antibodies in order to develop aptamer beacons that would detect exposure and an immune response to past CMV infection in a rapid, washless, homogeneous assay. Five known peptides from the anti-CMV hypervariable regions were used as targets for aptamer development and several 3D beacon models were developed with PatchDock and YASARA that appeared quite plausible, but only one of the aptamer beacons demonstrated a “lights on” fluorescence response with one of the CMV peptides [15]. Rigid 3D models predicted that all five systems should exhibit at least some beacon behavior, but empirical testing yielded only one successful system. 

#### 3.2.2. Lipopolysaccharide (LPS) Aptamer Beacons

**Figure 4:**
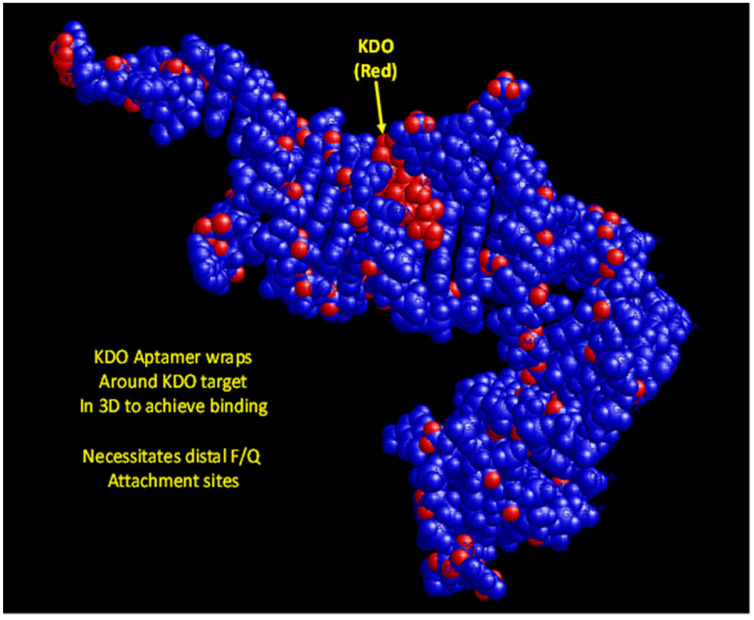
A space-filing 3D aptamer model visualized with RasMol software showing extremely tight binding to the core KDO antigen of LPS.

**Figure 5:**
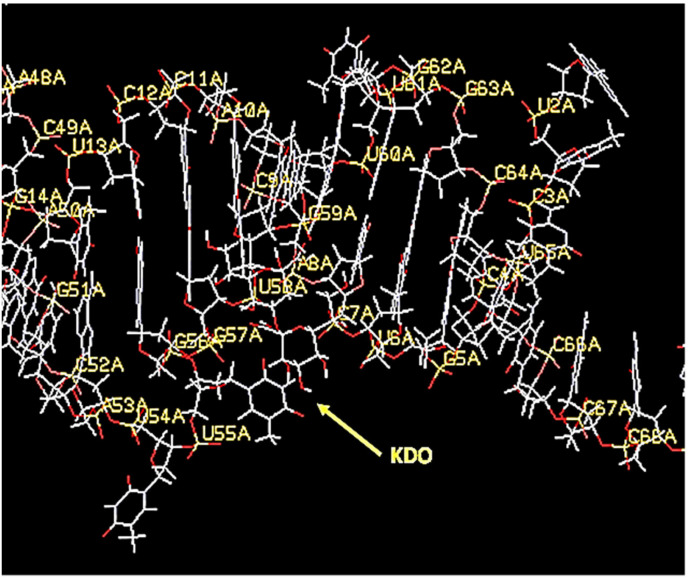
A 3D aptamer stick model in YASARA showing the close proximity of KDO core antigen to specific nucleotides seemingly within the 85–100 Å Förster distance, but when these bases were labeled with fluorophores and quenchers, no beacon responses were noted as a function of increasing KDO or LPS concentrations.

**Figure 6:**
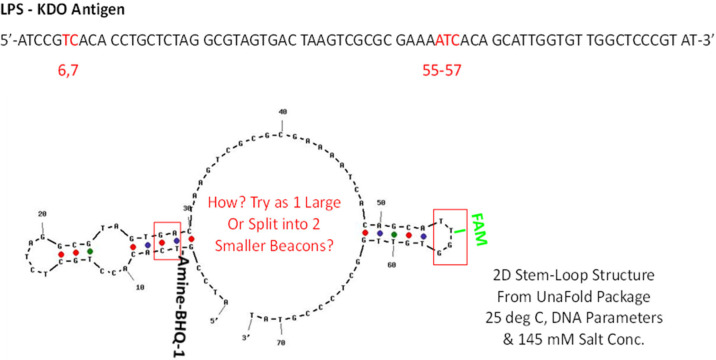
A 2D aptamer model generated by UnaFold showing the distal parts of the aptamer that appear to come together around KDO to achieve tight binding as shown in Figure 4 and Figure 5. Unfortunately, labeling these areas with fluorescein (FAM) and BHQ-1 still did not lead to a useful beacon.

**Figure 7:**
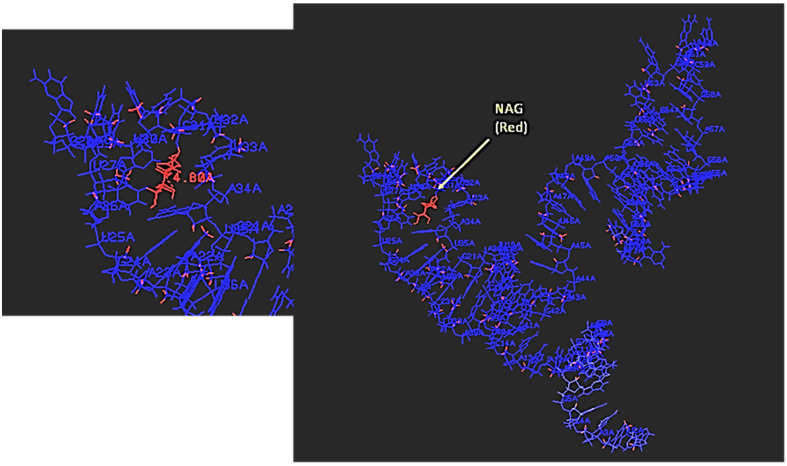
Static 3D model of NAG bound to the NAG-13F aptamer displayed in RasMol and showing the relative distances in Angstroms (4.8 Å for NAG as shown in the magnified left inset panel of the aptamer binding site, red text).

Medical science is in need of rapid detection of bacterial infections, especially in an age teetering on disaster due to multidrug-resistant bacteria. Thus, it is highly desirable to develop facile detection of urinary tract infections and sepsis. Consequently, the author developed aptamers against LPS [26] for Gram-negative infection diagnostics and aptamers against N-acetylglucoasmine (NAG) and polymers containing NAG such as chitin and peptidoglycan (PTG) for broader Gram-positive and Gram-negative and even fungal diagnosis [27,28]. However, to the author’s great frustration, none of the detailed 3D models have yet to produce useful FRET or a fluorescent beacon response. Figure 4, Figure 5, Figure 6 and Figure 7 illustrate details of the 3D LPS aptamer docking models including the location of the ketodeoxyoctanoate (KDO) core antigen from LPS in the aptamer model for other researchers to ponder and perhaps utilize as future successful beacon models. Figure 4, Figure 5 and Figure 6 are especially interesting since they seem to show distal parts of an aptamer that wraps around the core KDO region of LPS, but when labeled with fluorescein (FAM) and Black Hole Quencher (BHQ)-1, this model also failed to produce any beacon fluorescence response.

#### 3.2.3. N-Acetylglucosamine (NAG) and Chitin Aptamer Beacons

**Figure 8:**
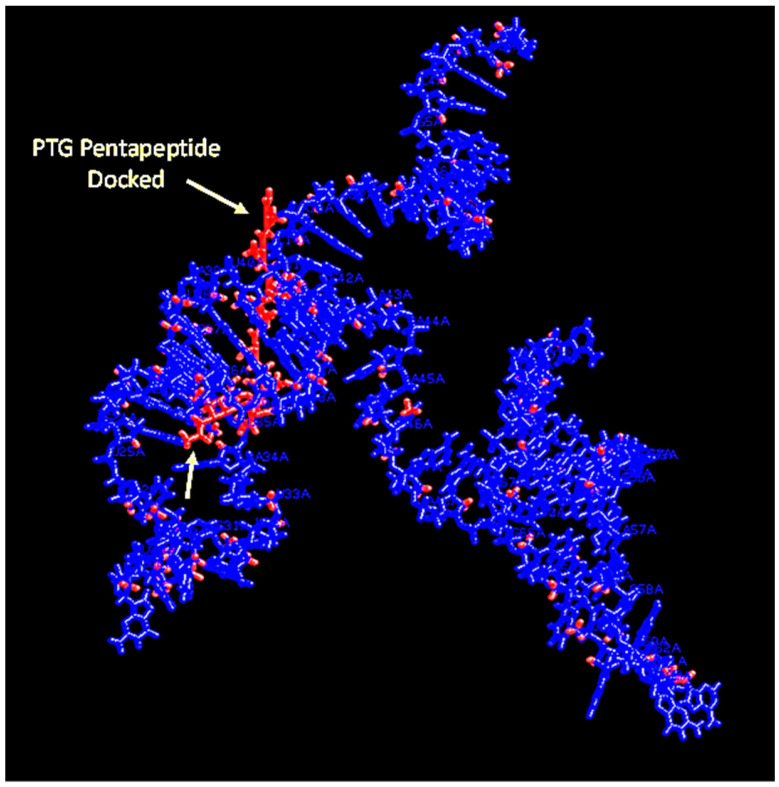
The NAG-13F aptamer bound to a short segment of peptidoglycan (PTG) pentapeptide shown in RasMol.

**Figure 9:**
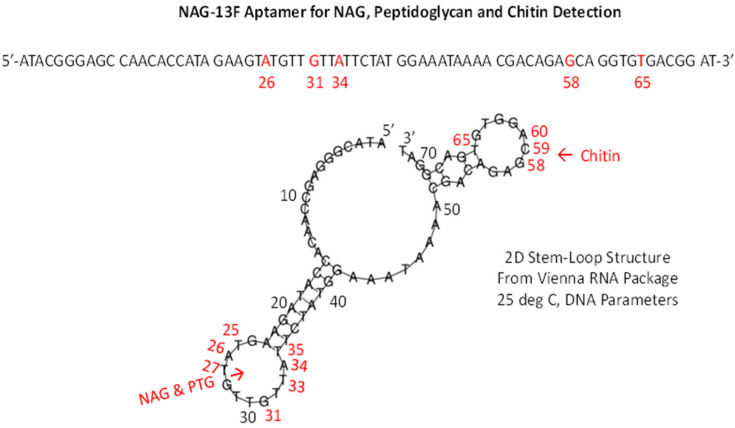
A 2D Vienna RNA-generated stem-loop structure of the NAG-13F aptamer showing the NAG and PTG binding site which is distal from the chitin-binding site according to PatchDock.

A similar frustration occurred when Bruno et al.’s top NAG aptamer [27] was studied for potential fluorescent beacon behavior to detect a broad array of bacteria and fungi. However, the interesting and serendipitous aspect of the best aptamer designated NAG-13F is that it also binds peptidoglycan (PTG; a polymer of repeating NAG and N-acetylmuramic acid or NAM dimers) proximal to the NAG binding site and chitin in a different section of the same aptamer at least according to the rigid 3D models (Figure 7, Figure 8 and Figure 9). While the NAG-PTG-chitin-binding aptamer never detected any fungi as a fluorescent beacon, it did bind these moieties in the cell wall of *Penicillium* mold as confirmed by aptamer-colloidal gold conjugate binding the chitin (a NAG polymer) seen in transmission electromicrographs in *Penicillium* cell walls [27] and significantly inhibited the cell wall synthesis and growth of *Saccharomyces* yeast by binding to chitin and inhibiting its cell wall incorporation in the growing yeast [28]. Thus, one could argue that the tight binding seen in the 3D docking models was confirmatory for other diagnostic and potential therapeutic aspects of the NAG-13F and related aptamers, but perhaps did not allow for FRET.

### 3.3. Semi-Successful

#### Crimean Congo Hemorrhagic Fever (CCHF) Virus Envelop Protein Binding

**Figure 10:**
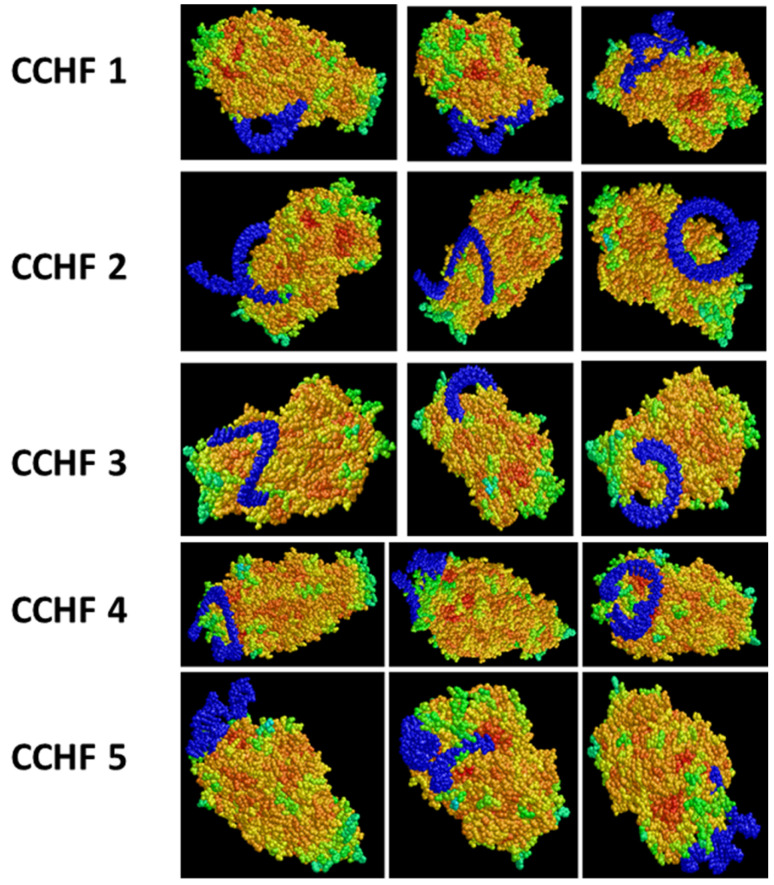
Three different views (presented in the three columns) of each of the top five highest affinity aptamers (blue) from ref. [29] bound to the full CCHF envelope protein [30].

Ten years ago, the author’s group published numerous aptamer DNA sequences that bound recombinant envelope proteins or associated peptide regions from several important arboviruses including CCHF for their diagnostic and therapeutic potential [29]. In an attempt to model and better understand any potential aptamer inhibitory effects on CCHF progression in vitro, Bruno et al. [29] set up static 3D aptamer–protein docking models using the top five aptamer DNA sequences that had been ranked based on affinity from ELISA-like binding assays [29] in conjunction with the full 1684 amino acid CCHF envelope protein (GenBank AHL45281.1 [30]) PDB file using PatchDock software. All five of the highest affinity aptamers demonstrated some ability to bind the exposed envelope protein as shown in Figure 10 below. 

At the time of writing this article, studies are still ongoing in a BSL-4 laboratory with viable the IbAr 10200 strain of the CCHF virus and the top five aptamer candidates. Optimal doses have not yet been determined for plaque reduction, but preliminary data seems to indicate that affinity is not always the best predictor of antiviral efficacy or host cell (SW-13 cell line)-entry blockage. Case in point, the blue aptamer “tightness” of binding (i.e., the amount of free space or contact between a given aptamer and the multicolored envelope protein in Figure 10) does not appear to be well correlated with plaque reduction efficacy in vitro, because the CCHF 2 aptamer has thus far demonstrated the best (up to 69%) reduction in plaque-forming units (pfus), but it shows some noteworthy spatial gaps between itself and the envelope protein’s surface in some of the views in Figure 10. The CCHF 4 and 5 aptamers which also performed well, but not as well as CCHF 2, in terms of pfu reduction (41% and 33.9%, respectively), appear to be bound more tightly to the envelope protein in Figure 10. Thus, in the end, efficacy against CCHF progression may boil down to where (locus on the envelope protein) the aptamer is binding and interfering with viral binding and host cell entry and may not be so dependent on how tightly the aptamer binds, if it binds less relevant regions or epitopes on the envelope protein. Therefore, overall, static 3D modeling appears somewhat successful or at least useful in this case, but one must not rely solely on binding proximity and “tightness” or on affinity predictions when attempting to predict how an aptamer may interfere with a receptor–ligand system.

### 3.4. Differences in Shape Complementarity Docking Programs

**Figure 11:**
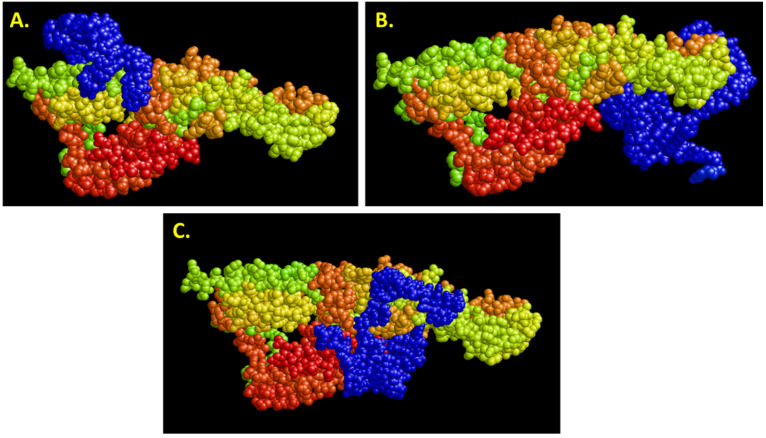
Analysis of binding sites on the Hanta virus envelope protein (multicolored) by the same blue DNA aptamer as predicted by the top scored docking model for (**A**) HDOCK, (**B**) ZDOCK and (**C**). PatchDock with clearly different optimal binding sites based on each program’s specific shape complementarity algorithm [31,32,33].

One fertile area for 3D docking model failures, or partial successes depending on how one views the results, is the choice of a shape complementarity docking program. While HDOCK, ZDOCK, PatchDock [31,32,33] and others claim to have optimal algorithms for finding the best receptor–ligand geometric fitting, it is nearly impossible to distinguish which actually gives the best fit. Assembling a jigsaw puzzle is more definitive, because one can at least see what the successful fitting of pieces looks like in the end result. However, as Figure 11 demonstrates using Bruno’s latest Hanta virus aptamer and envelope protein model, HDOCK, ZDOCK and PatchDock give very different aptamer binding locations when input with the same aptamer and protein –ligand data. Thus, one needs to be very careful and test the theoretical results empirically via an endpoint assay of X-ray crystallography, if possible to corroborate the theoretical model when visualized in RasMol or YASARA [34]. 

## 4. Conclusions

In the final assessment of this collection of static aptamer 3D model successes and failures, the primary problem appears to be the conversion of a 3D RNA structure that was folded as an RNA molecule into a DNA structure followed by “artificial” conversion back to DNA using Avogadro software instead of modelling it as a 3D DNA structure that was folded as DNA in the first place. At present, there may be no way around this problem, because one has to fold the aptamer as an RNA oligonucleotide using RNA Composer or Rosetta. Although the Rosetta suite of programs contains an application for studying 3D DNA structures and their docking with protein interfaces: (https://www.rosettacommons.org/docs/latest/application_documentation/design/rosetta-dna, accessed on 15 November 2022), it does not appear to enable DNA aptamer folding predictions that are sorely needed to improve static 3D aptamer-ligand modelling.

While there is some consideration of the impact of temperature, hydration and ionic strength inherent in secondary structure programs such as M-fold, UNAFOLD and Vienna RNA, there is no consideration of these environmental influences for the static 3D structures at present. Thus, when theory meets the real environmental world of various assay matrices, empirical results could vary greatly from theoretical predictions leading to poor prediction accuracy. 

Of course, editing RNA to convert it to DNA in Avogadro software is also tedious and susceptible to human errors. It must be checked and rechecked to ensure no errors in 2′-hydroxyl removal or uracil to thymine conversion (methylation) exist which could be serious in terms of overall incorrect binding models. There is also the question of which docking software to use (AutoDock, HDOCK, ZDOCK, PatchDock, etc. [31,32,33]) which could easily influence results as illustrated in Figure 11.

In the case of FRET or aptamer beacon failures, the Förster distance of less than 100 Å leaves little room for error in the theoretical placement of fluorophores and quenchers in the aptamer’s FRET “sphere.” Therefore, any vibration in the typically flexible aptamer, which is not accounted for in the static models, could lead to poor quenching and no apparent FRET or beacon fluorescence behavior as a function of analyte concentration. 

The author hopes that even the failures can be solved by others who may take up the projects described herein and approach them with MD analyses or future algorithms that may solve the stated problems. Regardless, these examples can provide didactic learning experiences that the wider scientific community may find somewhat beneficial and thus worthy of publication. 

## Data Availability

Not applicable.
